# Supplementary material for: A standard calibration method based on a symmetric resistance network matrix for galvanic logging instruments
Source: PLoS One. 2024 Apr 17;19(4):e0302032. doi: 10.1371/journal.pone.0302032 (PMC11023469; doi:10.1371/journal.pone.0302032)
Supplement: Attachment — Submitted filename: response to reviewers.docx [file pone.0302032.s002.docx]

25 January 2024

To Reviewer #1:

1. The sentences and grammar should be polished and modified by a native speaker. The current version is a little bit hard to understand;

Agreed. We have polished and modified the manuscript carefully.

2. It is better to give a workflow of the calibration method and describe the relationship between FEM and the resistance matrix method;

Agreed. A workflow is added and the relationship between FEM and the resistance matrix method is elaborated.

3. Please validate the accuracy of the new method in a layered medium.

Agreed. The accuracy of our method is validated through the comparison between different logging technologies in two real well.

To Reviewer #2:

1. It was well-written. However, the abstract shall be improved. Please include the important result in the abstract.

Agreed. We modified the abstract and added the key result.

2. All the variables, e.g., RB0, RB1, k0, Rx0, is1, es, etc., must be rewritten. They should be written in "equation" format.

Agreed. All the variables have been written in "equation" format.

3. All the mathematical expressions also should be rewritten in "equation" format.

Agreed. They have been rewritten in "equation" format.

4. Some variables in some equations were not defined. It is not consistent.

Agreed. We examined all variables and defined those without definition.

5. Equation (15): (Yn)(Phi) = ins. Based on Equations (13) and (14), the sizes of Yn and ins are 3x3 and 3x1, respectively, meanwhile the size of Phi is 4x1. The multiplication in Equation (15) involves the matrix multiplication of Yn and Phi to yield the result ins. Could you please explain in more detail how it works?

Agreed. This is our carelessness. We corrected the mistake and explained these Equation in more detail.

6. You did mention that "the value of each resistor can be obtained by inverting the formula (16)" (refer to line 186). Please include the inverse equation as your Equation (17).

Agreed. We added the inverse equation in the manuscript.

7. The way how you write nodes is not consistent. Please refer to lines 202 and 203 (without brackets) and lines 208 - 215 (with brackets). It is preferable to use the term "node" consistently before writing the number without brackets (e.g., "node 1"), instead of variations like "node (1)" or just "(1)" without the term "node".

Agreed. We rewrote all the nodes.

8. Could you please provide definitions for Y(1:3,1:3), Y(2:4,2:4), Y(1 3 4, 1 3 4) and Y(1 2 4, 1 2 4) in lines 208 - 215? While it is understandable that you are referring to rows and columns. However, it is preferable to define these terms first or present them in a more detailed manner.

Agreed. We noted them so that they make sense.

9. Please rewrite all the variables in Tables 1 - 10 in "equation" format.

Agreed. All the variables in Tables were rewritten in "equation" format.

10. Basically, this research paper is showing on how to derive a symmetric resistance network matrix by doing calibration with galvanic logging instruments. I think the title should include "calibration" and "galvanic logging instruments". Please consider this suggestion.

Agreed. The title has been modified.

11. I believe that Equation (28) should not be included in the manuscript. The equation is related to aspects of the work that have not been explained in the manuscript.

Agreed. Equation (28) has been deleted from the manuscript.

12. Statements in lines 281 - 283 and 291 - 293 are repeated.

Agreed. We removed duplicate statements.

13. In the abstract, you mentioned that the symmetrical resistance network can provide rapid calculation and improve the accuracy. However, your research work lacks results or proof supporting these statements.

Agreed. The description is not accurate. We edited these statements. As the nodes become more and more, the calibration circuit design is also more difficult to be equal to the real formation. This method of the manuscript is proposed as a standard calibration method to avoid the complex calibration design difficulty.

14. It is advisable to include an analysis or validation procedure in the manuscript. While the paper has demonstrated the calibration process leading to the development of a symmetric resistance network matrix, providing the significance of this matrix for galvanic logging applications in terms of accuracy or processing time would enhance the overall content in the manuscript.

Agree. We added two validation test to prove that the method have advantages.

15. All the best!!!

I appreciated your review and valuable suggestions. Best wishes to you!
