# Supplementary material for: A standard calibration method based on a symmetric resistance network matrix for galvanic logging instruments
Source: PLoS One. 2024 Apr 17;19(4):e0302032. doi: 10.1371/journal.pone.0302032 (PMC11023469; doi:10.1371/journal.pone.0302032)
Supplement: Attachment — Submitted filename: response to reviewers.docx [file pone.0302032.s003.docx]

18 March 2024

To Reviewer #1:

1. Please polish English writing. Some mistakes are existed. For example:

Table 2-9 ==> Tables 2-9.

Agreed. The manuscript has been polished and edited by a native English speaker.

2. All variables in the equation should be defined with their units added.

Agreed. We have added the units to all variables.

To Reviewer #2:

1.I have thoroughly reviewed the manuscript titled “A standard calibration method based on

symmetric resistance network matrix for galvanic logging instruments” and would like to

commend you on the comprehensive and meticulous manner in which you have addressed all

my queries and concerns. your careful attention to detail and the thoughtful revisions made have

significantly strengthened the overall quality of the paper. I would like to acknowledge that the

modifications you implemented have not only clarified several aspects of the research but have

also enhanced the overall coherence of the manuscript. Considering the thoroughness of your

revisions and the significant improvements made, I am pleased to recommend the acceptance

of your paper for publication. I believe that your research makes a valuable contribution to the

field, and I am confident that it will be well-received by the readership. Once again, thank you for

your diligence and commitment to advancing scientific knowledge. I look forward to seeing your

work published in PlosOne journal.

Thank you very much for your affirmation of the article. Wish you all the best!
